# The Effect of a Substrate Material on Composition Gradients of Fe-Ni Films obtained by Electrodeposition

**DOI:** 10.1038/s41598-019-57363-1

**Published:** 2020-01-23

**Authors:** Anna Maria Białostocka, Urszula Klekotka, Beata Kalska-Szostko

**Affiliations:** 1 Faculty of Electrical Engineering, Bialystok University of Technology, Wiejska 45D, 15-351 Białystok, Poland; 2 Institute of Chemistry, University of Bialystok, Ciołkowskiego 1K, 15-245 Białystok, Poland; 3 Institute of Chemistry, University of Bialystok, Ciołkowskiego 1K, 15-245 Białystok, Poland

**Keywords:** Chemistry, Engineering, Materials science, Physics

## Abstract

The electrodeposition of FeNi alloy films was performed galvanostatically in the sulfate solution (Fe^2+^/Ni^2+^ mass ratio 1:2) in order investigate their co-deposition mechanism. The FeNi layers were deposited at variable substrates (copper, brass, silver) under the same chemical (electrolyte composition) and electric plating (current density value) conditions. After applying various time, substrates and external magnetic field orientation, the quality of the obtained film was examined. The surface morphology and crystallographic texture variation were investigated by the Scanning Electron Microscope (SEM), Energy Dispersive X-ray Spectroscopy (EDX), Wavelength Dispersive X-ray Fluorescence (WDXRF) and Laser Confocal Scanning Microscope (LCSM). The anomalous co-deposition of iron group metals is evidently dependent on the substrate.

## Introduction

For years scientists have been expressing concern about the FeNi alloys. This is due to the fact that iron and nickel are notable for being components of metallic meteorites and dense metal cores of the planets such as Earth. The FeNi alloy makes up about 5.5% of the Earth’s core. Intermetallic compounds: Fe_3_Ni, Ni_3_Fe, and Ni_2_Fe are elements that build an extraterrestrial world. Scientists have been motivated to carry out research into the source of such amazing properties of the FeNi relationship and FeNi has been the subject of extensive experimental and theoretical research. Brenner was the first to put forward an idea of classifying alloy types. The principles he created govern the electrodeposition of the alloys and obtainment of the pure metals. The correctness and accuracy of the results of this process depend on the correct approach towards every stage in the whole process of crystallization. Max Volmer concepts of the crystallization’s steps controlled by charge transfer were described by Eq. (1)^1–3^.

1 $$\frac{j}{{j}^{o}}=\frac{{j}_{o}}{{j}^{o}}\left[\exp \left(\frac{{\alpha }_{a}F}{R\,T}\,\eta \right)-\left(\frac{c}{{c}_{0}}\right)exp\left(\frac{-{\alpha }_{c}\,F}{R\,T}\,\eta \right)\right]\,$$

where: $\eta =E-{E}_{r}$ - overpotential needed for the depositon of metals, c and c_0_ – concentration of the discharging species at the surface of the electrode and in the solution, j_o_ – exchange current density, α_a_ and α_c_ – anodic and cathodic transfer coefficient, j° – arbitrarily chosen unit current.

A further classification of metals divides them according to texture and grain size (Piontelli). The 20^th^ century yielded new influential theories (e.g., multiple nucleation and growth) and studies (e.g., surface diffusion, propagation, texture development, underpotential, and overpotential deposition). Metals are made of crystalline grains which are recognized by using Miller indexes (hkl) given to individual crystallographic forms^1–3^. Further calculations agreed with an up to date technique called “Tafel slope extrapolation”^4–6^. FeNi systems could be produced in the form of alloys, multilayers or nanowires by using several methods. The electrodeposition has been studied and presented in the literature by many authors. This mechanism has attracted scientists’ particular attention because of an anomalous co-deposition phenomenon^7^. Additionally, the electrodeposition technique allows to fabricate the alloys under normal conditions of temperature and pressure which are easy to control. This requires relatively cheap equipment, which implies low production costs and allows for nanostructuration. As a result, these parameters allow to obtain materials with specific, tailored properties of the grown nanomaterials. This “tailor-made” process positively affects the formation of crystals at the nano level. The creation and the growth of the nucleus is dependent on many electrodeposition parameters. This is extremely important due to the outcome parameters of the alloys, i.e. their mixture, level of granularity and roughness. The properties of the obtained layers depend primarily on basic conditions of the process itself: plating bath chemistry, bath pH, temperature, agitation (the chemical conditions), and the conditions at the cathode surface, current density, current characteristics, potential (the electric conditions)^8–11^. The nucleation depends on metal’s interaction with the substrate too. An interesting possibility for the contact-less control of the mass transfer is the magnetic field application^12–14^. The interaction with the external magnetic field (EMF) and electrochemical environment is known as the magnetohydrodynamic effect (MHD)^15,16^. It moves the electrolyte and changes the transport of the electroactive molecules to the electrode (diamagnetic ions, paramagnetic ions)^13,16^. The external magnetic field also influences the convection in the case of a mutual perpendicular position between the electric and magnetic field lines, which has been proven^13,17,18^.

Consumers require valuable properties of iron-nickel thin films which will be useful in different industry branches. There are new demands for alloy electrodeposition technologies. The role of FeNi coatings in the production of new nanodevices (MEMS, NEMS, FSMA, ULSI) is significant. It is also parallel to the development of nanotechnology. Thanks to their magnetic permeability and a small coercive force, FeNi constitutes magnetic components of products, for example, for reading and writing. Because of low thermal expansion coefficient, it also constitutes components of the television sets, the computer monitors, etc.^7,12^. Magnetic materials, such as nickel, iron, cobalt and their alloys, can replace ferromagnetic components in the future. Currently, wireless communication requires components in a small size, for example transformers, inductors. Applications of the “tailor-made” materials which will be very important in the future include high frequency magnetic applications, biomedical treatments and analysis, and high-density magnetic storage media. FeNi alloys have received great attention from scientists thanks to their interesting magnetic, photocatalytic, antioxidant, and biomedical properties. This article deepens the knowledge about the magneto-electro-deposition and in the field of material engineering, it will be the next step to “tailor” the new materials for next generations.

## Methods

Three different substrates were used to study the properties of the FeNi layers deposited electrochemically on them^19–22^. All solutions were prepared using deionized water and the following chemicals: 15% FeSO_4_·7H_2_O, 30% NiSO_4_·7H_2_O, 0.4% H_3_BO_3_ (POCH). In each experiment, a cylindrical glass beaker with a diameter of 0.045 m was filled with 2∙10^−5^ m^3^ of the electrolyte. The deposition container was placed centrally between two NdFeB permanent magnets (IBS Magnet) of approx. 1 T. A Pt plate with the dimensions: width-6∙10^−3^ m × height-5∙10^−3^ m × thickness-5∙10^−4^ m was a counter electrode. The working electrode (brass, silver, copper) had the following dimensions in all experiments: width-1∙10^−2^ m × height-2∙10^−2^ m × thickness-5∙10^−4^ m. The electrodes (anode and cathode) were placed centrally in the electrolyte and the distance between them was established at 2∙10^−2^ m. The magnetic field was created by two rectangular neodymium magnets of the following size: width-75∙10^−3^ m × height-50∙10^−3^ m × thickness-10∙10^−3^ m and the magnets were separated 55∙10^−3^ m from each other^23–25^. The magnets were magnetized perpendicularly to the magnet’s larger surface (75∙10^−3^ m × 50∙10^−3^ m) and B (II and _I_) was oriented like the x-axis in the Cartesian coordinate system. Magnetization vectors of both magnets were oriented anti-parallelly (II and _I_). The magnetic field distribution was measured along the largest magnet face with the gauge FH51 (Magnet-Physik). The value of the magnetic field strength varied according to the distance from the magnet and ranged from 80∙10^−3^ T to 20∙10^−2^ T (with an accuracy of ± 2%). In the area of conducting experiments, the magnetic field was uniform and the temperature was set at 294.15 ± 1 K. The process was performed galvanostatically by means of a potentiostat/galvanostat instrument (Matrix MPS-7163)^23–26^. The Scanning Electron Microscope (SEM) was used to investigate the surface morphology of the samples. The Energy Dispersive X-Ray (EDX) and Wavelength Dispersive X-ray Fluorescence (WDXRF) provided information about the alloy composition. The characteristics of the crystal structure of the probes were investigated using the X-Ray Diffractometer with Mo Kα radiation (wavelength 0.713067 10^−10^ m). Layers thicknesses were measured using the Laser Confocal Scanning Microscope – OLYMPUS LEXT 4000 with tenfold magnification.

### Preparation of the FeNi layers

**Figure 1:**
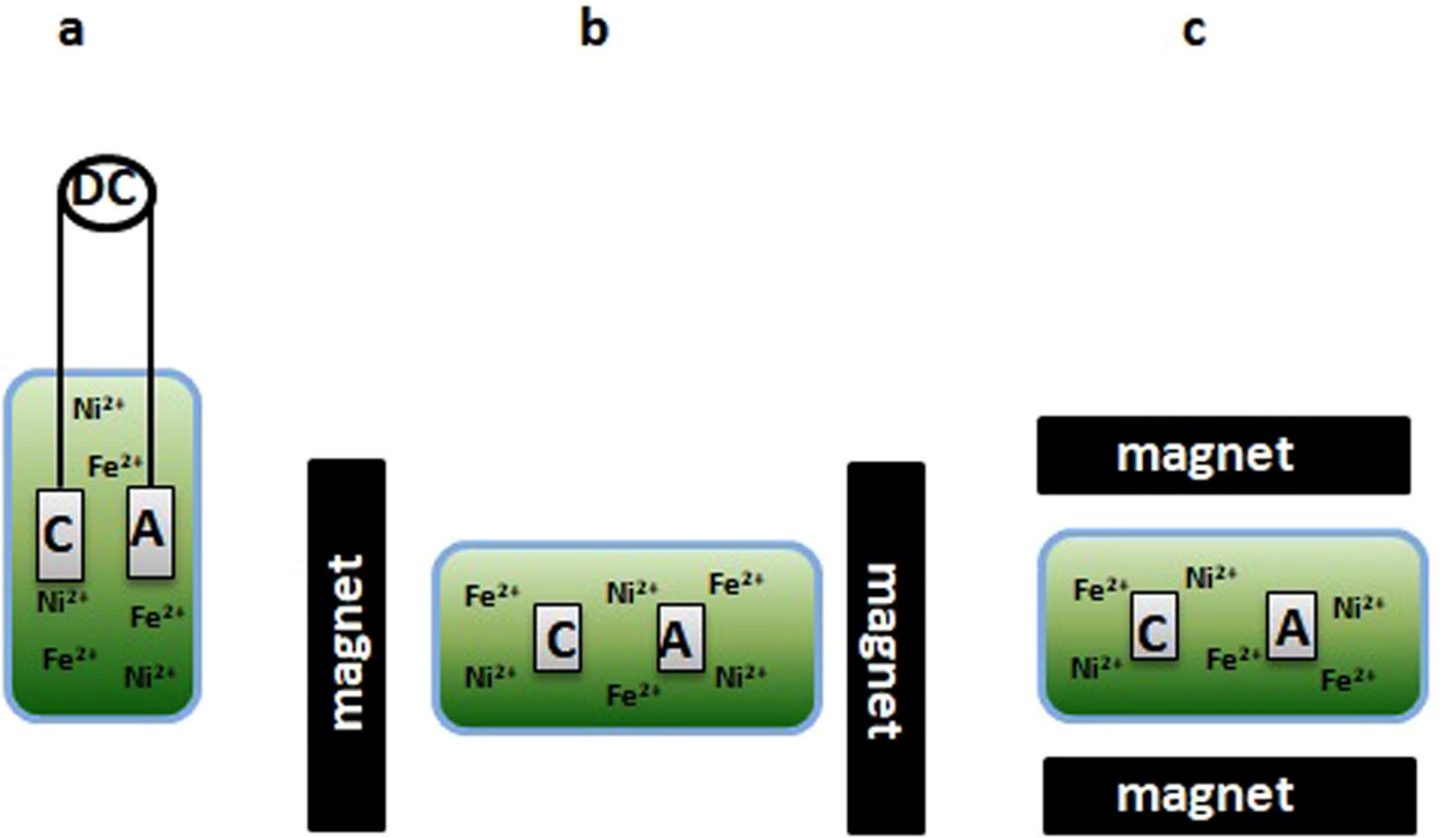
Schematic presentation of the deposition set-up (**a**) without the EMF (side view); (**b**) parallel orientation of the EMF (top view); (**c**) perpendicular orientation of the EMF (top view) in respect to the film plate.

The FeNi layers were deposited on electropolished plates using 50 mA/cm^2^ current density and in various time conditions (450, 900, 1800, 2700, 3600 s). During all experiments, an electrolytic environment with the same composition was used. The ratio of the nickel to iron ions was kept 2:1. The experiments were carried out in a specially designed measuring system. It enabled free adjustment of the source of the EMF to the electrodeposition system. In the final analysis, two mutual settings of magnetic field induction and electric currents were selected, parallel (II) and perpendicular (I_). Figure 1 shows the deposition set-up.

## Results

### Studies of the surface morphology

**Figure 2:**
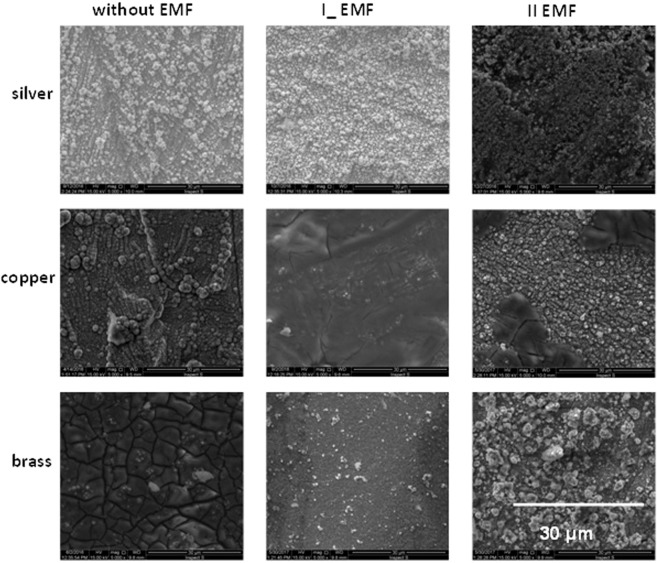
SEM images of the FeNi film on different substrate combinations: top) silver substrate; middle) copper substrate; bottom) brass substrate, magnification – 5000.

Figure 2 shows the influence of the EMF on the morphology of the electrochemically deposited FeNi alloy. The layers were deposited at the same time for 3600 seconds and under identical physicochemical conditions, and differed only in the substrate composition. After applying a silver substrate (without the presence of the magnetic field, I_ EMF), nucleation and growth took place simultaneously. The result was the presence of nucleation centers at various stages of development and of various sizes. Changing the setting of the external magnetic field to parallel resulted in the creation of micro vortices, and the nucleation mechanism changed to progressive. The layers deposited on the copper substrate without using the EMF looked similar to the silver deposit under the same conditions (without the magnetic field). Sludge on the Cu substrate after using the external magnetic field showed characteristic cracks and nodular shapes. FeNi morphology obtained on the brass substrate, similarly to the copper substrate, could be connected with the Fe content in the alloy. This took place during experiments conducted without the participation of the EMF and at II EMF. The third case (I_ EMF) was the reason for the creation of 3D growth centers scattered over the entire surface of the sample^25^.

**Figure 3:**
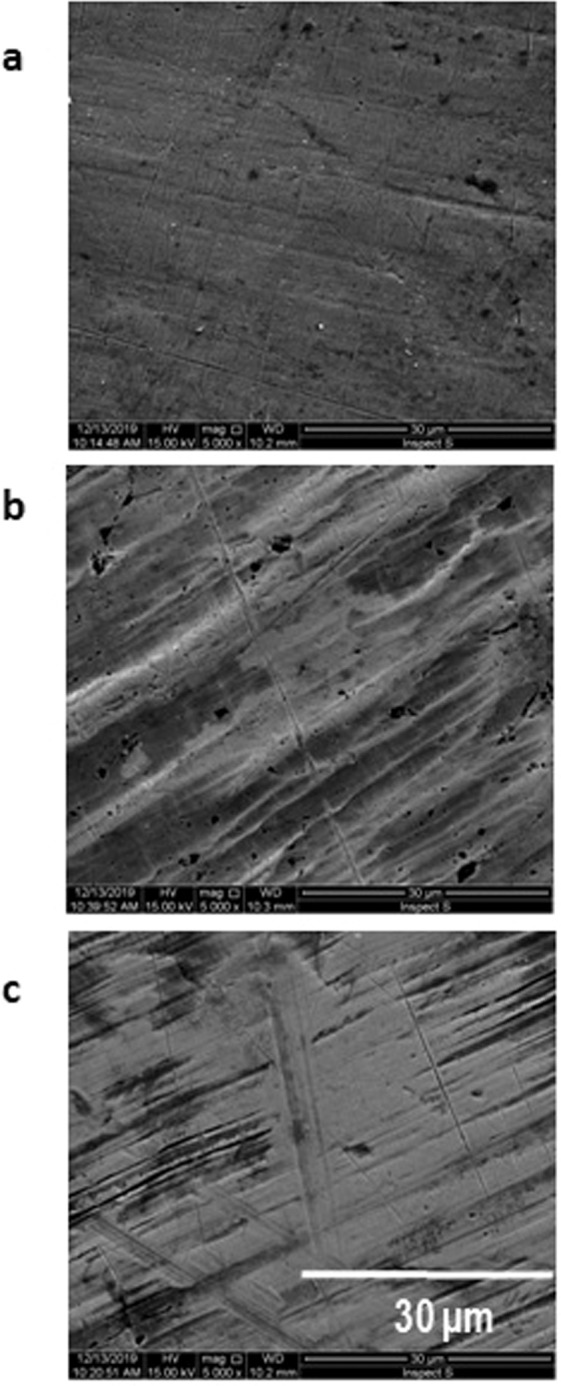
SEM images of the different substrate: (**a**) silver; (**b**) copper; (**c**) brass, magnification – 5000.

Figure 3 shows the morphology of the applied substrates. These morphologies show a similar view – the surface roughness.

**Figure 4:**
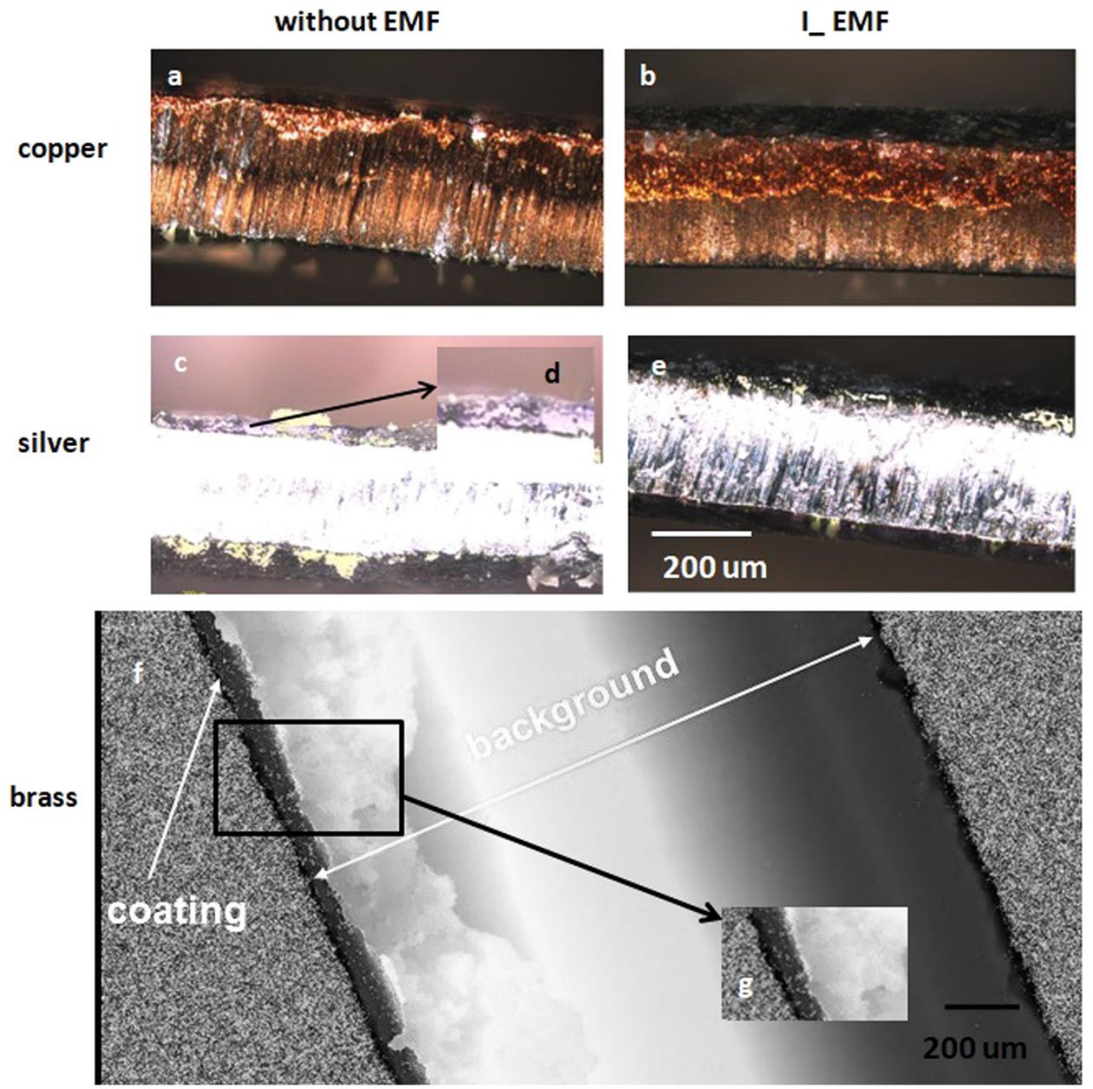
Cross-sections of the FeNi films on different substrate combinations with (right) and without (left) the EMF. top) FeNi film/Copper substrate; middle) FeNi film/Silver substrate; bottom) FeNi film/Brass substrate.

Figure 4 shows the cross-sections of the FeNi layers on all substrates. These cross-sections determine the degree of adhesion of the deposited layer to the substrate. This is especially important in the production for the needs of economy and industry.

The three substrates - silver, copper and brass - were selected for the deposition process. They exhibit similar magnetic features - diamagnetic properties. All the electrodeposited films had a high level of the adhesion. The results of the conducted experiments indicate the validity for the good adhesion of the deposited FeNi alloy to the substrates.

### Compositional studies

**Figure 5:**
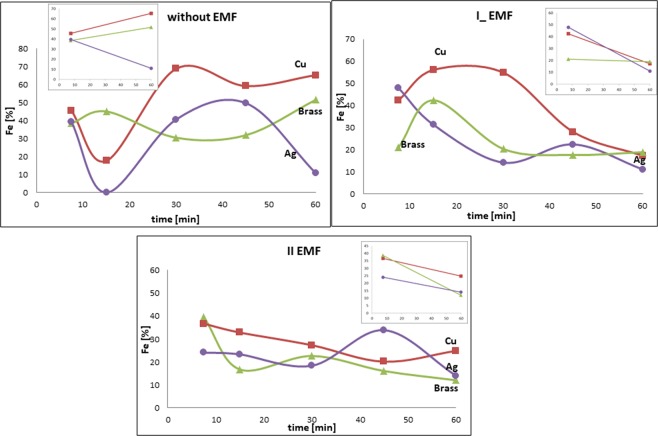
Time influence on the Fe content in the FeNi alloy using different substrates (copper – red squares, brass – green triangles, silver – purple circles), current density – 50 × 10^−3^ A/10^−4^ m^2^.

An undeniable connection between the iron content and the deposition base was found (Fig. 5): in the case of the deposition without the presence of the EMF, an increase in the iron content using a copper and a brass substrates, and decrease in the silver substrate. The situation changes after using the external magnetic field (EMF). In the layers deposited on the diamagnetic substrate (copper, brass), the change function of the alloy composition takes the opposite direction than earlier. The amount of iron shows a downward trend (Fig. 5). In the case of a silver substrate, the application of the EMF does not affect the composition of the alloy. The silver substrate did not have a major impact on the composition of the obtained alloy either without the presence of the external magnetic field or after its application (Fig. 5).

### Studies of the crystalline structure

**Figure 6:**
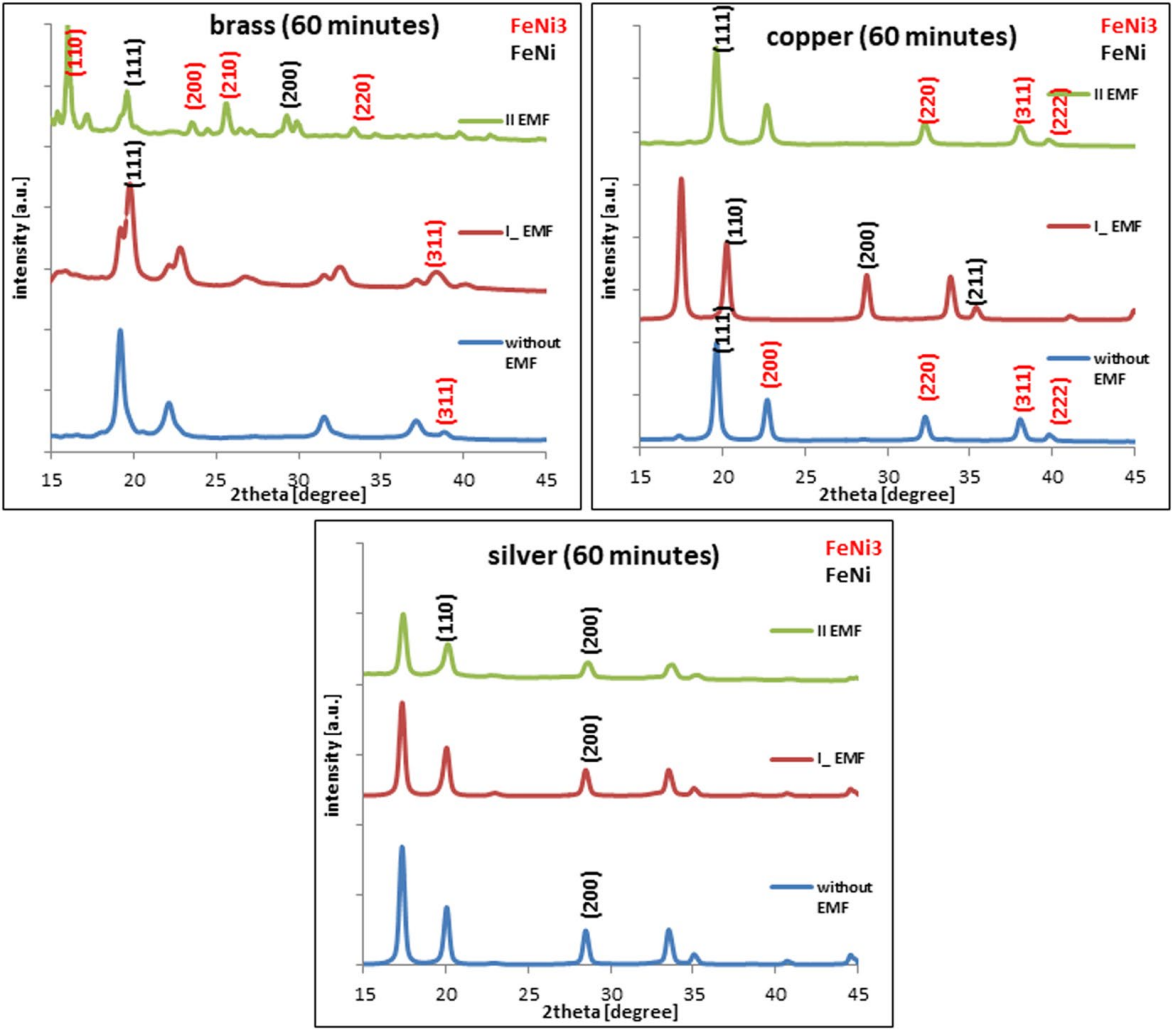
Diffraction patterns of the FeNi electrodeposited films: left – brass; middle – copper; bottom – silver, current density – 50 × 10^−3^ A/10^−4^ m^2^, time of the deposition – 3600 s.

The crystalline structure of the alloy deposited on the copper substrate shows a dependence on the orientation of the external magnetic field (Fig. 6). The perpendicular and parallel settings of the EMF significantly activate the bcc and fcc structures respectively. This is in line with the obtained percentage composition of FeNi. The addition of zinc in the brass substrate changes the crystal structure of the alloy. In the case of applying a parallel field, it is already a mixture of FeNi and FeNi_3_ with the domination of FeNi_3_ – fcc structure. The silver substrate does not change the crystal structure of the iron-nickel alloy and is typical of FeNi.

## Discussion

The application of the EMF significantly affected the morphology of the layers deposited on the silver substrate. The layer formation process stabilized and the MHD effect was visible. The result (3D growth centers) was increased by the mass transport to already existing nuclei (I_ EMF). The cracks and nodular shapes visible in the sediment on the copper substrate after the application of EMF are related to the decrease in iron content in the alloy, released in the form of iron hydride (Fig. 2). The reason for this was a high value of the hydrogen diffusion coefficient in this location. The cracks and nodular shapes also occur in the brass substrate and again, this is related to a diffusion coefficient of the brass component-zinc^26,27^.

The deposition without the external magnetic field results in the instantaneous nucleation mechanism. After application of the I_ EMF, significant changes occurred on all of these substrates. The EMF set in parallel way changed the mechanism of nucleation and growth process on the brass substrate from instantaneous to progressive. The density of occurring nuclei has decreased and their size has increased. Cauliflower-shaped regions with a well developed structure are visible. Sediment on other substrates (silver, copper) has retained its character with granular structure-like shapes (Fig. 2). Therefore, the influence of the substrate on nucleation mechanism and subsequent growth process of the deposited layer is confirmed.

The situation with similar views of the substrates surface roughness does not influence the early nucleation and layer growth phases (Fig. 3).

The degree of adhesion of the mentioned layers is closely related to crystallographic coherence at the boundary of the deposited layer and substrate. Good cohesion and adhesion is mainly related to the evolution of hydrogen. Its release on the surface causes gaps between the sediment and the ground (Fig. 4C,D,F,G). This is directly related to the hydrogen diffusion coefficient. The copper and silver have a similar value of the coefficient (Cu – 11.3 × 10^−7^ m^2^∙(s)^−1^, Ag – 10 × 10^−7^ m^2^∙(s)^−1^). It is much higher than in the case of iron (1.0 × 10^−8^ m^2^∙(s)^−1^) and nickel (1.8 × 10^−7^ m^2^∙(s)^−1^). In this case, hydrogen atoms can diffuse into the substrate in a simple manner. The result is crystallographic coherence and the layer match between the sediment and substrate^19,28^.

After analysing the alloy composition (Fig. 5), it may be concluded that the composition is also associated with the value of the diffusion coefficient of the substrate’s building material, which has already been reported by scientists. The value of this coefficient for silver is lower than for other substrates. The diffusion process is particularly important in the case of electrodeposition in the outer magnetic field. When the EMF is used, diffusion-controlled mass transport is important, and the thickness of the diffusion layer is reduced. The lack of the EMF’s effect on the deposition on the silver substrates and the visible effect of EMF on deposition on the copper and brass substrates are illustrated.

The investigation revealed that the crystalline structure of the fabricated films depends on the substrate used. Diffractograms confirmed the influence of the crystal structure of the substrate only in the case of the copper and brass. The silver substrate did not influence the crystal structure of the deposit^19,20,29–33^. The effect of the substrate on the alloy’s composition and its crystal structure is the correlation between the magnetic nature and the diffusion coefficient of the substrate.

## Conclusions

The electrodeposition has been used to obtain the FeNi thin films on the three different substrates. For each of them, the use of an external magnetic field resulted in morphological changes in the sediment. Generally, an iron-nickel layer with a fcc structure will favorably grow on a substrate with the same structures, respectively (fcc on fcc.). The diffractograms registered for the alloys embedded without the EMF presence resulted in the creation of homogeneous and mixed structures. On the diamagnetic substrates (silver) only the crystal structure of FeNi was obtained while the composition of the second substrate (brass) was influenced by the admixture of copper and zinc in the composition. In its core, the FeNi and FeNi_3_ crystals were found. The influence of the magnetic field in terms of the composition was observed only in the case of two substrates – the copper and brass. The alloy composition in the case of silver substrate did not show any downward changes after the EMF use.
