# Author Correction: The Effect of a Substrate Material on Composition Gradients of Fe-Ni Films obtained by Electrodeposition

**DOI:** 10.1038/s41598-020-64609-w

**Published:** 2020-05-01

**Authors:** Anna Maria Białostocka, Urszula Klekotka, Beata Kalska-Szostko

**Affiliations:** 1 Faculty of Electrical Engineering, Bialystok University of Technology, Wiejska 45D, 15-351 Białystok, Poland; 2 Institute of Chemistry, University of Bialystok, Ciołkowskiego 1K, 15-245 Białystok, Poland; 3 Institute of Chemistry, University of Bialystok, Ciołkowskiego 1K, 15-245 Białystok, Poland

Correction to: *Scientific Reports* 10.1038/s41598-019-57363-1, published online 23 January 2020

The Acknowledgements section in this article is incomplete.

“This work was partially financed by the EU fund as part of the projects: POPW.01.03.00-20.034/09 and POPW.01.03.00-20-004/11, and supported by a research subsidy for 2020 assigned to teamwork. The authors would like to thank Professor Dominik Dorosz and Professor Piotr Żabiński for their help.”

should read:

“This work was partially financed by the EU fund as part of the projects: POPW.01.03.00-20.034/09 and POPW.01.03.00-20-004/11, and supported by a research subsidy of the Institute of Automation, Electronics and Electrotechnology Bialystok University of Technology for 2020 assigned to teamwork. The authors would like to thank Professor Dominik Dorosz and Professor Piotr Żabiński for their help.”

In addition, the legend of Figure 6 is incorrect:

“Diffraction patterns of the FeNi electrodeposited films: left – brass; middle – copper; bottom – silver, current density – 50 × 10^−3^ A/10^−4^ m^2^, time of the deposition – 3600 s.”

should read:

“Diffraction patterns of the FeNi electrodeposited films: left - brass; right - copper; bottom - silver, current density – 50 × 10^−3^ A/10^−4^ m^2^, time of the deposition – 3600 s.”

